# Factors influencing the decision that women make on their mode of delivery: the Health Belief Model

**DOI:** 10.1186/s12913-015-0931-z

**Published:** 2015-07-20

**Authors:** Alice Yuen Loke, Louise Davies, Sau-fun Li

**Affiliations:** School of Nursing, The Hong Kong Polytechnic University, 11 Yuk Choi Rd, Hung Hom, Hong Kong

**Keywords:** Mode of delivery decisions, Cesarean section, Vaginal birth, Health Belief Model

## Abstract

**Background:**

Childbirth is regarded as an important life event for women, and growing numbers of them are making the choice to give birth by Caesarean Delivery. The aim of this study was to identify the factors influencing the decision that women make on their mode of delivery, underpinned by the Health Belief Model.

**Methods:**

This was a cross-sectional study. Hong Kong Chinese women aged 18–45, who were pregnant or had given birth within the last three years were recruited. The participants were asked to complete a structured self-administered questionnaire consisting of 62 questions.

**Results:**

A total of 319 women were recruited, of whom 73 (22.9%) preferred to have a cesarean section delivery (CD). The results showed that women preferred CD because they were concerned about being pregnant at an advanced age, were worried about labor pain and perineum tearing, wanted to have a better plan for maternity leave, had chosen an auspicious date to deliver, and perceived that CD is a more convenience way to deliver. The perceived benefits and severity of a vaginal birth (VB), and the perceived benefits, severity, and cues to action of CD, affected the decision to undergo either a VB or CD.

**Conclusions:**

The data indicated that the constructs of the Health Belief Model – perceived benefits, perceived severity, and cues to action – affect the decision that women make on their mode of delivery. This research indicates that there is value in designing educational programs for pregnant women to educate them on the benefits, risks, and severity of the two different modes of birth based on the constructs of HBM. This will enable women to be active participants in choosing the mode of birth that they believe is right for them.

## Background

Historically, the natural process of Vaginal Birth (VB) has been viewed as the unquestioned mode of birth, whereas Caesarean Section Delivery (CD), which involves an operative incision, has been perceived as a risky procedure designed for women with medical indications [57]. With advances in reproductive technology, an increase in the number of CDs has been observed in recent years [9]. There has also been a shift in the attitudes, so that it is no longer unusual for couples to request a CD [30]. “Caesarean Delivery on Maternal Request” (CDMR) refers to a primary CD performed prior to labor in the absence of medical indications, where women are choosing for themselves their preferred mode of birth [57].

Childbirth is regarded as an important life event for women, and growing numbers of them are making the choice to give birth by CD. The escalating CDMR rate is associated with the perception of women that CD is the safer mode of birth [51]. However, the evidence to support this belief is limited.

Rates of CDMR seems to be increasing worldwide, more so in some countries than others. According to a report by the National Institute of Health [38], approximately 4–18% of all babies in the United States were born by CDMR in 2004. Overall, CDs in the United States have increased from 22.9–32.8% between 2000 and 2010 [31]. In 2011, it was reported that 8% of the increase in CDs at a major hospital in the United States was attributable to CDMR [3].

Much higher rates of CDs are observed in Asian countries. In the urban regions of China, a CD rate of 54.1% was reported in 2008 [54] followed by Taiwan at 35.2% in 2007 [8]. Between the years 1998 to 2008 in China, the rate of CDs in rural regions rose from 3.6 to 23.6%, and that for urban regions rose from 19.9 to 54.1% [54]. There is evidence of an increase in the CDMR rate from 0.8% in 1994 to 20% in 2006 [24]. According to a territory-wide obstetric audit in Hong Kong, elective CDs for non-medical indications increased from 5.5% of all CDs in 1994 to 16.7% in 2004 [19]. A more complete picture of the number of babies being delivered by CDMR will help to determine whether rates of CDs are indeed increasing.

### Factors influencing maternal preference of mode of birth

There are various factors influencing a woman’s choice of mode of birth. Demographic factors and an individual’s expectation of childbirth have a bearing on her decision-making process. Others are previous birth experience, potential complications arising from the mode of birth, and concerns over the health and safety of mother and baby [41].

### Application of the health belief model on the maternal choice of mode of birth

In the present study, the Health Belief Model (HBM) was adopted as a conceptual framework, to provide a sound theoretical basis for understanding the factors that influence women’s childbirth decisions. The HBM can specify the relationship between health-related beliefs/factors and maternal behaviors, which can help in predicting the possibility of a woman choosing a particular mode of birth. Using this model, mode of birth and maternal choice and its determining factors can be explored within the five domains of the HBM, namely: perceived susceptibility, perceived severity, perceived benefits, perceived barriers, and cues to action [23].

### Perceived susceptibility

Perceived susceptibility is a person’s belief in his/her vulnerability to some medical condition. The more that a person believes he/she is at great risk, the more likely that person is to adopt a particular health-related behavior to minimize such risk [23]. For instance, a negative experience in a previous birth could affect a woman’s preference for a particular mode of birth in subsequent births, due to the belief that the negative experience could occur again [40].

### Perceived severity

Perceived severity is defined as one’s belief in the intensity of the medical condition and its undesirable outcomes [23]. If it is believed that there are very serious or intolerable complications associated with a specific mode of birth, women are more likely to express a preference for an alternative method of delivery, so as to reduce their risk.

For both VB and CD, the most severe complications are maternal and neonatal mortality [47]. A global survey by the World Health Organization between 2004 and 2008 reported the risks of maternal mortality and morbidity in CD without medical indications [28]. The risks due to CD were three times greater than those for VB, including in the areas of maternal mortality, admission to an intensive care unit, the need for a blood transfusion, and the need to carry out a hysterectomy or internal iliac artery ligation. It was noted that CD can have several negative consequences on maternal health, including adverse outcomes related to anesthesia, adhesion formation, and uterine rupture [47]. Neonatal respiratory depression secondary to maternal anesthesia has also been identified as a risk associated with CD [10].

However, VB is not without risk. Maternal complications associated with VB include pelvic organ prolapse, prolonged labor, and perineal trauma [1, 41]. For the neonate, there is also an increased risk of contracting infections such as Hepatitis C, HIV, and HPV during vaginal birth from maternal to neonate transmission [47].

### Perceived benefits

Perceived benefits are defined as one’s belief that outcomes can be positively affected by engaging in a particular health behavior [23]. The advantages of maternal and fetal health and a sense or anticipating fulfillment and satisfaction of sociocultural beliefs have been identified as important factors in maternal decision making.

When considering the perceived benefits for the health of childbearing women, it has been noted that in a number of countries women associate VB with a greater number of benefits than CD. Women in Singapore (91.5%), Turkey (89%), and the USA (42%) believed that VB offers a faster recovery, earlier discharge, and the absence of a CD scar [7, 10, 27, 42].

When focusing on neonatal health, nearly 60% of women believed that VB is safer for the baby [10]. Women also reported that VB enables earlier bonding with their baby and early initiation of breastfeeding.

In comparison, a fear of labor (50%) and repetitive vaginal examinations (23%) were underlying reasons why women showed a preference for CD [10]. This was supported by women identifying tocophobia (an intense fear of labor contractions), prolonged labor, fetal distress, and the perineal trauma associated with VB as reasons for why they planned to have a CD [5, 32, 40, 52]. Women also took into consideration the advantages of CD in maintaining genital appearance (24%), facilitating tubal ligation (20.6%), and minimizing sexual dissatisfaction (0.8%) following delivery [5, 10].

From another perspective, women also perceived CD as more convenient, allowing them to better plan their maternity leave. It is also of significance that within the Chinese culture, some women strongly believe that an auspicious time of birth is vital to a person’s lifelong fate and destiny [21]. It has been noted that the birth rate in the year of the dragon in the Chinese zodiac, a particularly auspicious year, rises [55].

### Perceived barriers

Perceived Barriers refers to an individual’s perception of the difficulties stopping them from following a specific health-related behavior [23]. The desire to choose VB is hindered by existing medical contraindications. There are some medical contraindications of VB for mothers, including pelvic disproportion, pre-eclampsia, severe cardiovascular disease, diabetes mellitus, active genital herpes, HIV infection, and multiple pregnancies [4, 29, 48]. On the other hand, the medical contraindications for babies include fetal malpresentation, fetal malformation, cord prolapse, and macrosomia [39, 49].

In Hong Kong, CDMR is only available in the private sector, as public hospitals will not permit this practice. The cost of a CDMR in the private sector ranges from $23,000 to $66,800 (all figures are quoted in Hong Kong dollars) or even higher, which is far more expensive than a VB [18, 20]. Given that the median monthly household income in Hong Kong was about $18,000 in 2010 [22], this implies that women of lower financial status cannot afford the CDMR plan in the private sector. Both public hospital policy and low financial status could act as barriers to choosing a CD. Studies have shown that insurance coverage is a vital element in the maternal choice of delivery, with studies conducted in Australia and Chile indicating that insurance coverage encourages women to attend private hospitals and hence encourages CDMR [12, 37].

### Cues to action

Cues to action refer to the factors that help individual make health-related decisions [23]. Advice from relatives, friends, health care professionals, as well as an awareness of the rights of women are crucial factors guiding the maternal decision on delivery method.

Women’s beliefs and attitudes towards a particular mode of delivery are strongly influenced by the stories and advice that they hear from relatives and friends [5, 11]. Women were driven to an alternative mode of delivery after hearing negative stories about a particular mode increasing concern that they might have the same experience when they gave birth [33, 46]. In addition, the pregnant woman might also worry if there is a family history of poor obstetric outcomes [43]. Advice from health care professionals such as midwives and doctors very much influences a woman’s understanding of a particular delivery mode and her preference for it [13].

Other than advice from others, some women perceived that they should have their own right to decide the mode of delivery [25]. This is a major reason why CDMR rates are increasing worldwide [41].

### Significance of the study

The issue of maternal preference for a particular mode of birth is complex. The aim of this study is to examine the perceptions of Hong Kong women towards CD and VB, as well as their priorities when they are considering their mode of birth. With insight into women’s attitudes and preferences on mode of birth, midwives and obstetricians can better support women by providing appropriate information during pregnancy, enabling them to make an informed choice and take an active part in the decision-making process.

## Methods

### Study design

This was a descriptive cross-sectional study. A structured self-administered questionnaire was used to collect information from Hong Kong women of childbearing age on their preference for mode of birth and the factors that influenced this preference, guided by the five constructs of HBM.

### Study setting and sampling

The target population of this study was married Hong Kong women aged 18–45. The criteria for inclusion in this study were women who are (1) permanent Hong Kong residents and able to read and write Chinese, and (2) pregnant or had given birth within the past three years. Women who had medical indications for CD were excluded.

A convenient sampling strategy was adopted to recruit the potential participants, on the basis of their availability and willingness to participate. Women were approached as they were entering or leaving public hospitals under the jurisdiction of the Hospital Authority or from Maternal and Child Health Centers across various districts of Hong Kong, including those located in the eastern part of Hong Kong, Kowloon Center, Kowloon West, and the New Territories. Self-administered questionnaires were distributed to those who agreed to fill them out. The questionnaire took an average of approximately 10-15mins to complete.

All potential participants were invited to take part in the study in August and September of 2013. The sample size was determined based on the general rule that 5–10 subjects need to be recruited for each item in a questionnaire [17]. As the questionnaire contains 47 items (excluding demographic questions), a total of 235–470 subjects needed to be recruited.

### Questionnaire development

A questionnaire was developed specifically for this study based on the studies of Chong and Mongelli [7], Pang et al. [40], Buyukbayrak et al. [5], and Dursun et al. [10], which identified a maternal preference for VB or CD.

The questionnaire is made up of four parts. Part A collects the socio-demographics data of the participants. Part B was designed to collect information on a woman’s preference on mode of birth (without financial and medical considerations), sources of information, and the factors influencing their preference.

Parts C and D of the questionnaire contain different statements about the different perceptions relating to VB and CD correspondently based on the five constructs of HBM: perceived susceptibility, perceived benefits, perceived severity, perceived barriers, and cues to action. Previous studies discussing the influencing factors and attitudes toward VB and CD formed the basis for the questionnaire items in these two parts [5, 7]. The women were asked to indicate their level of agreement towards the statements using a four-point Likert Scale (strongly disagree, disagree, agree, and strongly agree). This even-point scale requires the respondent to give either a positive or negative response, as no option for a neutral response is provided.

There were 21 items in Part C, measuring the perceived susceptibility (2 items), perceived benefits (10 items), perceived severity (5 items), perceived barriers (1 item), and cues to action (3 items). There were 26 items in Part D, measuring the perceived susceptibility (2 items), perceived benefits (15 items), perceived severity (3 items), perceived barriers (2 items), and cues to action (4 items). For the calculation of mean scores, “strongly agree” was assigned a score of 2 and “agree” a score of 1, whereas no score was accorded to the scales “disagree” or “strongly disagree”. A higher score signifies a stronger perception of a specific construct.

### Validity and reliability

A panel of experts that included an obstetrician, a midwife, and an obstetric nurse was invited to validate the questionnaire. Most of the questionnaire items were evaluated by the three experts as appropriate and relevant to the study, with the Content Validity Index equal to 0.94. Minor amendments were made to the wording and order of the questionnaire to achieve a more logical layout. A pilot study was then conducted before the commencement of the study in August 2013 among 30 women to test the comprehensibility of the items and to establish the reliability of the questionnaire. Further amendments were made to unfamiliar phrases that required clarification from the women in the pilot study. The overall Cronbach’s alpha of the pilot study was calculated to be 0.896, indicating that the instrument has a high level of internal consistency.

### Ethical considerations

Ethical approval was obtained from the Human Subjects Ethics sub-committee of the Hong Kong Polytechnic University prior to the commencement of the study. All eligible women were recruited on a voluntary basis. Potential participants were provided with a written information sheet stating the purpose of the study and details regarding adherence to the Privacy Ordinance, supplemented with an explanation if needed, before their written consent was obtained. The returned questionnaires were anonymous and could not be identified by the researchers. Only the authorized researchers could access data for analysis.

### Statistical analysis

The data that were obtained were entered and analyzed using IBM Statistical Package for Social Sciences (SPSS Version 19.0, USA). Descriptive statistics and a Chi-square test were used to identify and compare the demographic information, influencing factors, and the five constructs of the HBM between two preference groups. An unpaired *t*-test was used to determine whether there were any statistically significance differences regarding perceived benefits, severity barriers, and cues to action between women who prefer VB and CD. Finally, logistic regression was used to determine whether maternal characteristics and scores derived from constructs of HBM are predictors of maternal preference on mode of delivery. The significance level (α) was set at 0.05.

## Results

During the data collection period 340 Hong Kong women who fulfilled the criteria for inclusion were invited to participate in the study. A total of 326 women consented to take part in the study, for a response rate of 95.9%. Of these, 7 returned questionnaires were incomplete and excluded, leaving 319 valid questionnaires for analysis.

### Characteristic of the study participants

The questionnaires from the 319 respondents were analyzed in this report. The demographics of the women who participated are shown in Table 1. The majority of the women were between 26–35 years of age (71.5%), with 18% in the age group of 36–45. Two-thirds were pregnant (66.7%) and one-third (33.3%) of the women had given birth within the past three years. The majority (79%) were born in Hong Kong. Over half (52%) were recruited from the New Territories. Well over half had attained a tertiary level education (58%) and held full-time employment (65.5%). Only 8.2% were health professionals. Nearly one third (30.4%) had a monthly household income of more than HK$40,000, and about half had a monthly income of HK$10,001–30,000.

**Table 1:** Demographics of women of childbearing age in Hong Kong: comparison between women who prefer a vaginal birth (VB) and a cesarean delivery (CD) (n = 319)

	Total	Prefer VB	Prefer CD	P-value
	(n = 319)	(n = 246)	(n = 73)	
	n (%)	n (%)	n (%)	
Age group				0.008**
18–25	33 (10.3)	25 (10.2)	8 (11.0)	
26–35	228 (71.5)	185 (75.2)	43 (58.9)	
36–45	58 (18.2)	36 (14.6)	22 (30.1)	
Place of birth				0.488
Hong Kong	252 (79.0)	198 (80.5)	54 (74.0)	
Outside Hong Kong (China and Asia)	67 (21.0)	48 (19.5)	19 (26.0)	
Place of residence				0.470
New Territories	166 (52.0)	130 (52.8)	36 (49.3)	
Kowloon	125 (39.2)	97 (39.4)	28 (38.4)	
Hong Kong Island	28 (8.8)	19 (7.7)	9 (12.3)	
Educational level				0.016*
Tertiary or above	185 (58.0)	153 (62.2)	32 (43.8)	
Secondary school	133 (41.7)	92 (37.4)	41 (56.2)	
Primary school	1 (0.3)	1 (0.4)	0 (0.0)	
Employment status				0.446
Homemaker	88 (27.6)	66 (26.8)	22 (30.1)	
Full time employment	209 (65.5)	165 (67.1)	44 (60.3)	
Part-time employment	22 (6.9)	15 (6.1)	7 (9.6)	
Women’s occupation				0.016*
Non-health related	293 (91.8)	221 (89.8)	72 (98.6)	
Health related:	26 (8.2)	25 (10.2)	1 (1.4)	
Physician		1 (0.4)	0 (0.0)	
Nurse		15 (6.1)	1 (1.4)	
Allied health (PT/OT)		9 (3.6)	0 (0.0)	
Monthly household income				0.240
HK$ 0–10,000	17 (5.3)	11 (4.5)	6 (8.2)	
HK$ 10,001–20,000	79 (24.8)	59 (24)	20 (27.4)	
HK$ 20,001–30,000	78 (24.5)	57 (23.2)	21 (28.8)	
HK$ 30,001–40,000	48 (15.0)	37 (15)	11 (15.1)	
Over HK$ 40,000	97 (30.4)	82 (33.3)	15 (20.5)	
Sources of information on modes of delivery:				
Obstetrician	196 (61.4)	150 (61.0)	46 (63.0)	0.753
Nurses	71 (22.3)	56 (22.8)	15 (20.5)	0.689
Relatives	105 (32.9)	84 (34.1)	21 (28.8)	0.390
Friends	86 (27.0)	66 (26.8)	20 (27.4)	0.923
Previous birth experience	82 (25.7)	65 (26.4)	17 (23.3)	0.590
Internet / books	81 (25.4)	72 (29.2)	9 (12.3)	0.03*

* = P <0.05, ** = P < 0.01

Overall, without financial and medical considerations, 246 out of the 319 women (77.1%) indicated a preference for VB, while the remaining 73 women (22.9%) preferred CD. The demographic characteristics of the respondents were further compared according to the maternal preference of mode of birth using Chi-square tests (Table 2). Those who preferred CD, when compared with those who preferred VB, were more likely to be in the advanced maternal age group of over 36 years old (30.1% vs. 14.6%), less likely to have received a tertiary level education (43.8% vs. 62.2%), and more likely to be in a non-health related profession (98.6% vs. 89.8%). There were statistically significant differences between the two groups of women in terms of age (p = 0.008), level of education (p = 0.016), and occupation (p = 0.016).

**Table 2:** Considerations when making the decision on mode of delivery: Comparison between women who prefer a vaginal birth (VB) and a cesarean delivery (CD) (n = 319)

	Prefer VB	Prefer CD	P-value
	(n = 246)	(n = 73)	
	n (%)	n (%)	
Baby’s factors			
Health of the newborn	209 (85.0)	39 (53.4)	<0.001***
Birth trauma to the newborn	52 (21.1)	24 (32.9)	0.039*
Respiratory trauma to the newborn	20 (8.1)	10 (33.7)	0.152
Newborn’s birth presentation	24 (9.8)	13 (17.9)	0.059
Large baby	23 (9.3)	16 (21.9)	0.004**
Twins/triplets	5 (2.0)	5 (6.8)	0.038*
Maternal factors			
Maternal health	180 (73.2)	33(45.2)	<0.001***
Advanced age for childbirth	17 (6.9)	22 (30.1)	<0.001***
Labor pain	47 (19.1)	58 (79.5)	<0.001***
Unsightly abdominal scars	63 (25.6)	5 (6.8)	0.0010**
Worry about tearing of the perineum	18 (7.3)	19 (26.0)	<0.001***
Possible anal/urinary incontinence due to VD	6 (2.4)	19 (26.0)	<0.001***
Possible better sexual satisfaction	1 (0.4)	12 (16.4)	<0.001***
Social factors			
Natural way of delivery	122 (49.6)	13 (17.8)	<0.001***
Faster/more convenient method of delivery	7 (2.8)	18 (24.7)	<0.001***
Certainty about the timing of the delivery	8 (3.3)	20 (27.4)	<0.001***
Better planning for maternity leave	8 (3.3)	20 (27.4)	<0.001***
Better planning for paternity leave	4 (1.6)	11 (15.1)	<0.001***
Medical insurance coverage	11 (4.5)	2 (2.7)	0.511
Choosing an auspicious date	0 (0.0)	14 (19.2)	<0.001***
Women should have the right to choose	14 (5.7)	14 (19.2)	<0.005**

A Chi-square test was used to compare the factors that childbearing age women would take into consideration when making their decision

* = P < 0.05, ** = P < 0.01, *** = P < 0.001

Over half of the women (61.4%) reported that they had been provided with information on the different modes of delivery by relatives (32.9%) and friends (27%), and to a lesser extent from nurses (22.3%).

### Factors influencing mode of birth

Women were asked to indicate, if given a free choice on mode of birth without financial and medical considerations, all of the factors that they would consider when making a decision on VB or CD. Table 2 shows the factors that these women took into consideration Table 2.

The most commonly cited reasons for preferring VB were “concern for health of the newborn” (85%), followed by “concern for maternal health” (73.2%), and “being a natural way of delivery” (49.6%). The main reasons for preferring CD were “avoidance of labor pain” (79.5%), “concern for the health of the newborn” (53.4%), and “concern for maternal health” (45.2%). Statistically significant differences between the two groups of women preferring VB or CD were demonstrated for the most popular influencing factors (P < 0.001).

### Constructs of the health belief model

The women’s perceptions of VB and CD were assessed based on the five constructs of HBM: perceived susceptibility, benefit, severity, barriers, and cues to action. A comparison was then made between women who preferred the two different modes of delivery.

Table 3 shows the women’s perceptions of VB according to the five constructs of HBM, and the comparison between the two groups of women. Among women who expressed a preference for VB, only a small number considered themselves susceptible to common complications such as “painful labor” (n = 15) and postpartum hemorrhage (n = 4) due to VB. Women who expressed a preference for VB in comparison with those who preferred CD were more likely to perceive the benefits of VB. The top three benefits of VB were deemed to be: “a normal or natural process” (100 vs. 75.4%), “allows early breastfeeding” (94.7% vs. 76.7%), and “faster recovery” (98.4% vs. 64.4%). Statistically significant differences were found for all three items, with p = <0.001. Women who preferred VB were significantly less worried about the perceived severity of “fetal injuries with the baby bore vaginally” (39.4% vs. 90.4%) and “perineal tears” (43.9% vs. 87.7%). Fewer of them possess insurance for CD in a private hospital (30.9% vs. 35.6%), but there was no statistically significant difference. More of them reported that “advice from relatives/friends” (83.7%) and “health care professionals” (82.6%) were their cues for action.

**Table 3:** Constructs of HBM relating to women’s perceptions of vaginal birth (VB): Comparison between women who prefer a vaginal birth (VB) and a cesarean delivery (CD) (n = 319)

Women’s perception of vaginal birth (VB)	Prefer VB	Prefer CD	P-value
	(n = 246)	(n = 73)	
	n (%)	n (%)	
Perceived susceptibility			
Painful labor process	10 (0.04)	5 (0.07)	0.455
Postpartum hemorrhage	1 (0.004)	3 (0.04)	0.034*
Perceived benefits			
VB is a normal/natural process	246 (100.0)	55 (75.4)	<0.001***
Allows early contact with newborn after delivery	230 (93.5)	48 (65.8)	<0.001***
Allows early breastfeeding	233 (94.7)	56 (76.7)	<0.001***
Shorter hospital stay	228 (92.7)	45 (61.6)	<0.001***
Faster recovery after delivery	242 (98.4)	47 (64.4)	<0.001***
No unnecessary surgical wound pain	225 (91.4)	41 (56.2)	<0.001***
No need for an operation and anesthesia	231 (93.9)	48 (65.7)	<0.001***
The fate of my baby is determined by nature	193 (78.4)	28 (38.4)	<0.001***
Less costly	209 (84.9)	55 (75.3)	0.056
Covered by insurance/hospital authority	122 (49.6)	29 (39.7)	0.138
Perceived severity			
Risk of fetal injuries when the baby goes through the vaginal canal	97 (39.4)	66 (90.4)	<0.001***
Risk of mother-to-child transmission of infectious agents during vaginal birth	131 (53.2)	63 (86.3)	<0.001***
Worry about perineal tears due to vaginal birth	108 (43.9)	64 (87.7)	<0.001***
Afraid of damage to the pelvic floor due to vaginal birth	84 (34.2)	55 (75.4)	<0.001***
Concern over having urinary/anal incontinence if a vaginal delivery is performed	90 (36.6)	52 (71.2)	<0.001***
Perceived barriers			
Carry insurance coverage for CD in private hospitals	76 (30.9)	26 (35.6)	0.447
Cues to action			
Healthcare professionals advise VB	203 (82.6)	52 (71.2)	0.034*
Relatives/friends advise VB	206 (83.7)	45 (61.6)	<0.001***
Have heard negative stories from relatives/friends about their cesarean delivery	78 (31.7)	24 (32.8)	0.851

* = P < 0.05, *** = P < 0.001

Table 4 shows the women’s perceptions of CD according to the five constructs of HBM, and the comparison between the two groups of women. It was revealed that women considered themselves prone to “abdominal wound infections” (n = 4), and “a long recovery time” (n = 3) due to CD. Women who expressed a preference for CD, in comparison with those who preferred VB, were more likely to identify the benefits of CD. The top benefits were being able to “avoid prolonged labor” (100% vs. 75.2%), “prevent labor pain (97.2% vs. 69.9%), “reduction of fear induced by prolonged labor and fetal injuries” (91.8% vs. 50.7%), and being “a faster and more convenient way of delivery” (91.8% vs. 51.7%). On all of these items, statistically significant differences between the two groups of women, with p < 0.001. These women, compared with their counterparts, were also less likely to perceive the severity of using anesthesia during CD (61.6% vs. 92.7%, P = <0.001), less worried about “uterine scar ruptures” (34.2% vs. 50.9%), and “the formation of scar adhesion” (41.1% vs. 55.7%). They also were more likely to consider the restrictions placed by public hospitals on CDMR to be a barrier to the use of CD (57.4% vs. 83.6%). “Advice from health care professionals” (72.6%), “friends/relatives” (45.2%), “have heard negative stories about VB” (46.6%), and “a family history of difficulty in childbirth” (37.0%) were all cues to action for those who expressed a preference for CD.

**Table 4:** Constructs of HBM relating to women’s perceptions of cesarean delivery (CD): Comparison between women who prefer a vaginal birth (VB) and a cesarean delivery (CD) (n = 319)

Women’s perceptions of cesarean delivery (CD)	Prefer VB (n = 246)	Prefer CD (n = 73)	P-value
	n (%)	n (%)	
Perceived susceptibility			
Abdominal wound infection	4 (0.02)	0 (0.0)	0.329
Long recovery time	1 (0.004)	2 (0.03)	0.140
Perceived benefits			
A faster/more convenient method of delivery	127 (51.7)	67 (91.8)	<0.001***
A trendy/modern method of delivery	109 (44.3)	60 (82.2)	<0.001***
Less fear of prolonged labor and fetal injuries	124 (50.7)	67 (91.8)	<0.001***
Avoid pain induced by repetitive vaginal examinations	111 (45.2)	55 (75.4)	<0.001***
Avoid the necessity of inducing labor	123 (50.0)	59 (80.8)	<0.001***
Prevent labor pain	172 (69.9)	71 (97.2)	<0.001***
Avoid prolonged labor	185 (75.2)	73 (100)	<0.001***
Preserve sexual function and genital appearance	94 (38.2)	43 (58.9)	0.002**
Minimize potential sexual dissatisfaction	66 (26.9)	36 (49.3)	<0.001***
Allows tubal ligation after CD	94 (38.2)	32 (43.8)	0.388
Allows better planning of maternity leave	168 (68.3)	58 (79.4)	0.065
Allows better planning of paternity leave	165 (67.1)	55 (75.4)	0.180
Avoids the uncertainty of the timing of the delivery	185 (75.2)	60 (82.2)	0.214
Can select an auspicious date to deliver my baby	162 (65.9)	49 (67.1)	0.840
Year, date, time, and weekday of birth affect one’s fate	70 (28.4)	34 (46.6)	0.004**
Perceived severity			
Concern over the anesthesia complications of CD	228 (92.7)	45 (61.6)	<0.001***
Afraid of uterine scar ruptures if a cesarean delivery is performed	125 (50.9)	25 (34.2)	0.013*
Afraid of adhesion formation if a cesarean delivery is performed	137 (55.7)	30 (41.1)	0.028*
Perceived barriers			
Extra cost of CD out of own pocket	125 (50.8)	38 (52.1)	0.852
Cannot choose CD in a public hospital	141 (57.4)	61 (83.6)	<0.001***
Cues to action			
Healthcare professionals advise CD	132 (53.6)	53 (72.6)	0.004**
Relatives/friends advise CD	40 (16.2)	33 (45.2)	<0.001***
I heard negative stories from relatives/friends about their vaginal delivery	72 (29.3)	34 (46.6)	0.006**
I have a family history of difficult births	44 (17.9)	27 (37.0)	0.001***

** = P < 0.01, *** = P < 0.001

### Comparison of mean scores between the two preference groups

The mean scores of the constructs of the HBM were analyzed and compared between the two groups of women. As perceived susceptibility was only reported by a few women and there was no statically significant difference between the VB or CD groups, no further analysis was conducted on this construct.

A comparison of the mean scores for the remaining four domains of HBM between the two groups women is given in Table 5. The comparison of the four constructs of the perceptions of VB showed that women who preferred VB had a significantly higher mean score on perceived benefits (12.61 vs. 7.52) and a lower mean score on perceived severity (2.54 vs. 5.40 than those who preferred CD (P < 0.001).

**Table 5:** The constructs of HBM between the two groups of women who prefer a vaginal birth (VB) or a cesarean delivery (CD)

Mean ± SD			
	Prefer VB (n = 246)	Prefer CD (n = 73)	P value
Perceived benefits (VB)	12.61 ± 3.577	7.53 ± 4.285	<0.001 ***
Perceived severity (VB)	2.45 ± 2.334	5.40 ± 2.419	<0.001 ***
Perceived barriers (VB)	0.35 ± 0.548	0.45 ± 0.668	0.167
Cues to action (VB)	1.50 ± 0.951	1.33 ± 1.131	0.208
Perceived benefits (CD)	9.63 ± 6.035	15.66 ± 5.697	<0.001 ***
Perceived severity (CD)	2.63 ± 1.548	1.60 ± 1.431	<0.001 ***
Perceived barriers (CD)	2.08 ± 1.572	2.36 ± 1.513	0.180
Cues to action (CD)	1.22 ± 1.241	2.05 ± 1.373	<0.001 ***
Logistic regression (Forward Wald): Predictive constructs for preferring CD		Odd Ratio (95% CI).	P-value
Perceived benefits (CD)		1.127 (1.028–1.235)	0.011*
Perceived benefits (CD)		0.677 (0.480–0.955)	0.026*
Perceived benefits (VB)		0.712 (0.625–0.810)	<0.0001 *
Perceived severity (VB)		1.565 (1.264–1.938)	<0.0001 *

The higher mean scores for the four constructs of the perceptions of CD indicated that women who preferred CD had a significantly higher mean score on perceived benefits (15.66 vs. 9.63), a lower mean score on perceived severity (1.6% vs. 2.63), and a higher mean score on cues to action (2.05 vs. 1.22) than those who preferred VB (P < 0.001).

### Constructs of HBM as predictors of a maternal preference for cesarean delivery

A logistic regression analysis was conducted to assess whether maternal characteristics and the constructs of HBM were predictive of a maternal preference for CD (Table 5). Variables that had been found in a previous analysis to be statistically significant, including the demographic characteristics of the respondents as well as the HBM constructs, were entered as independent variables for analysis. The preference for CD as the mode of birth was set to be the dependent variable.

As shown in Table 5, a statistically significant link to a maternal preference for CD was found for those who perceived that CD offered higher benefits (OR = 1.565, P = 0.0001) and less severity (OR = 0.677, P0.026), and that VB offered less benefits (OR = 0.712, P < 0.0001) and more severity (OR = 1.127, P = 0.011).

## Discussion

The age-specific fertility statistics reported by the Hong Kong Census and Statistics Department in 2013 [6] show that the majority of births occur among women in the age group of 25–34. In this study, the majority of women of childbearing age were between 26–35 years of age, showing that the sample in this study is comparable to that for Hong Kong in general. In addition, the largest proportion of the sample were living in the New Territories (52%), followed by Kowloon (39.2%) and Hong Kong Island (8.8%). These percentages are consistent with the geographical distribution of the Hong Kong female population, with the majority of women (51.9%) living in the New Territories in 2011 [6]. It is concluded that the sample in this study is representative of women of childbearing age in Hong Kong.

In this study, the majority of the Hong Kong women indicated their preference for VD (77.1%) over CD (22.9%). This percentage is similar to that found in other countries of Asia, with a high percentage of women in South Korea (96.9%), Singapore (95.1%), and Turkey (84.1%), preferring VB as their mode of birth [5, 7, 27]. A high preference for VB, at 89%, was also reported in a study conducted in North Carolina, USA [44]. The reported 22.9% preference rate for CD is lower than in the urban regions of China (54.1%) [54], Taiwan (35.2%) [8]. However, at 22.9% the preference rate for CDs expressed by the women in this study is somewhat higher than the rate for elective CDs for non-medical indications in Hong Kong in 2004, at 16.7% [19]. That percentage also exceeds the rate of 10–15% for CDs considered optimal by the World Health Organization [53]. This indicates that an increasing number of Hong Kong women prefer to give birth by CD.

This study found that the majority of women (61.4%) received information on mode of birth from their obstetrician. However, it has been revealed that most of the information given out by obstetricians relates to the birth procedure rather than to the possible risks and benefits of the different modes of delivery [13]. This study also found that women identified nurses (22.3%) as less likely to have been a source of information related to birth than their own relatives (32.9%) and friends (27%). This result shows that obstetricians and nurses are not providing sufficient information. More education on health concerns and modes of birth is needed if childbearing women are to make an informed decision on mode of birth.

### Characteristics of women preferring two different modes of delivery

The results of this study indicate that age, level of education, and occupation are significant correlates for women’s preference on mode of birth. A preference for CD was associated with advanced maternal age. Previous studies have speculated about the relationship between advanced maternal age and the likelihood of CD, in that pregnant women of advanced age have been shown to hold a strong belief that their advanced age puts them and their baby at risk during labor and delivery, due to the physiological factors related to aging [53]. A study in Taiwan also revealed that older women worried whether their baby would be able to pass safely through the vaginal canal [5]. This concern has led to the belief that CD is safer mode of delivery for pregnant women of advanced age.

Women with higher levels of education were found to be more likely to choose VB as their preferred mode of birth. This finding is inconsistent with other reports indicating that more educated women would choose CD as the mode of delivery [56]. Further studies are required to determine the reasons for this difference, such as the level of knowledge on childbirth held of these women, or social and economic differences between the studied populations.

The women in this study who were health professionals were more likely to choose a vaginal birth. This is consistent with other studies that showed that the majority of obstetricians and nurses preferred to have a VB due to their consideration that VB is a normal and natural low-risk life event, and to their better understanding of the complications of CD [2, 26, 50].

### Considerations when making decisions on mode of delivery

The most common concerns of women who preferred VB were over maternal health (73.2%) and the health of the baby (85%). This is consistent with the results from other studies. Studies have reported that the majority of women considered VB to be a safer mode of birth for the mother (81.7%) and the neonate (72.8%) [42]. The majority of women prefer VB because it allows for an earlier discharge from hospital and for the mother to recover more quickly [5]. The risk of surgery and anesthetic drugs passing to the neonate in CD is also a consideration for women who prefer VB [10].

On the other hand, the most common reasons for women to request a CD was to avoid labor pain (79.5%), concern for the health of the newborn (53.4%), and worry about potential birth trauma (32.9%) and respiratory trauma (33.7%) to the newborn from a vaginal birth. Labor is often thought of as one of the more painful events in human experience and women are fearful of experiencing pain during labor [7].

As a vaginal birth is regarded as “the most natural process of birth,” women who want to have a natural birth prefer VB (49, 6%). This is also an important reason given by the nearly 90% of women in other studies who choose VB [10, 27]. However, women who preferred CD expressed a wish for certainty about the date of the birth (27.4%), and thus to be better able to plan for maternity leave (27.4%). This is perhaps understandable for contemporary women who have their own career, with 19.2% of the participants indicating that they wanted to have the right to choose the mode by which their baby would be delivered.

### The HBM constructs and maternal preference on mode of birth

There were significant differences between women who preferred VB and CD in their perceptions of the benefits and severity of different modes of birth. Women who preferred VB perceived the benefits of VB as being that it is a normal and natural process (100%), recovery is faster after delivery, it allows for earlier breastfeeding, and no unnecessary surgery and anesthesia is involved in the process. These women were also less worried about fetal injuries during vaginal birth than those who preferred CD. Women who preferred CD believed that by opting for this process they would be able to avoid prolonged labor (100%), labor pain (97.2%), and fetal injuries, as well as have a fast and convenient delivery. For women who preferred CD, advice from health professionals, negative stories of VB from relatives and friends, as well as a family history of difficult births were the cues for action.

Women who preferred VB had a significantly higher mean score on the perceived benefits and a lower score on the perceived severity of VB than those who preferred CD. Women who preferred CD had a significantly higher mean score on the perceived benefits and a lower score on the perceived severity of CD, and a higher mean score on cues to action than those who preferred VB. These results are consistent with those of other studies. Studies have confirmed that perceived benefits are a predictive factor of delivery preference [14, 34, 35]. Women weighed the considerations of the maternal/fetal benefits and complications of a particular mode of birth [10, 45], demonstrating the importance of these constructs of HBM in decision making.

Cues to action indicated that advice from professionals played an important role in the maternal decision on mode of birth, especially for CD. It has been reported that advice from physicians is an important influence on women in their choice of mode of birth [16]. A study has shown that only 5% of women continued to attempt a VB when they perceived that their obstetrician held an unfavorable attitude towards VB [15]. Some midwives have actually been reported to encourage women to undergo a CD in order to protect their pelvic floor and reduce the risks of developing urinary or fecal incontinence [36].

The logistic regression analysis of the HBM demonstrated that the constructs of perceived benefits, perceived severity, and cues to action were significant correlates of the maternal preference on mode of birth. These factors should be considered when designing educational interventions to help women make the decision on the mode of delivery that is most appropriate for their needs.

### Limitations

This study is not without limitations. First, the participants of this study were women recruited outside public hospitals, or Maternal and Child Health Centers. Those who visited private hospitals were not recruited, which could lead to bias in sample recruitment. Second, this study explored only the perception of women towards the two types of modes of delivery but not the actual delivery mode they had or will be taken. Third, this was a cross-sectional study, and thus there is limited information on how women form a preference for mode of delivery, perception changes, and the dynamic decision making process throughout pregnancy in their preference for mode of delivery.

## Conclusions

The results of this study provide a better understanding of the prevalence and the factors influencing the choice of mode of delivery among childbearing women in Hong Kong. However, a longitudinal study is needed to identify if women change their perception and choice on mode of delivery during pregnancy or after delivery.

Although more women in this study preferred VB, there is evidence of a growing preference for CD. While age, level of education, and involvement in a health-related profession influenced the decision made by the women in this study, the perceptions of the benefits and severity of the different modes of delivery were the most important considerations. There is also evidence that advice from health professionals plays an important role in the maternal decision on mode of birth. There is a need for comprehensive information on the benefits and severity of the different modes of delivery, instead of just the birth procedures. Women of childbearing age should have a right to receive comprehensive and unbiased information from health professionals so that they can make an informed choice on the mode of birth that is most suitable for them.
